# The association between albumin corrected anion gap and ICU mortality in acute kidney injury patients requiring continuous renal replacement therapy

**DOI:** 10.1007/s11739-022-03093-8

**Published:** 2022-09-16

**Authors:** Lei Zhong, Bo Xie, Xiao-Wei Ji, Xiang-Hong Yang

**Affiliations:** 1 Soochow University, Soochow, 215000 Jiangsu China; 2 Department of Intensive Care Unit, Huzhou Central Hospital, Affiliated Central Hospital, Huzhou University, Huzhou, 313000 Zhejiang China; 3 Department of Intensive Care Unit, Zhejiang Provincial People’s Hospital, Hangzhou Medical College, Hangzhou, 310000 Zhejiang China

**Keywords:** Albumin corrected anion gap, Acute kidney injury, Continuous renal replacement therapy, Mortality

## Abstract

The relationship between albumin corrected anion gap (ACAG) and mortality in acute kidney injury (AKI) patients who received continuous renal replacement therapy (CRRT) has not been investigated in any previous studies. This study aimed to investigate the relationship between ACAG at CRRT initiation and all-cause mortality among these patients in the intensive care unit (ICU). Patients diagnosed with AKI and treated with CRRT in the ICU from the Medical Information Mart for Intensive Care-IV version 1.0 (MIMIC IV) database and Huzhou Central Hospital were retrospectively enrolled. Participants were divided into two groups: the normal ACAG group (12–20 mmol/L) and high ACAG group (> 20 mmol/L). The Kaplan–Meier method and log-rank test were used to compare the survival rate between the two groups. Restricted cubic spine (RCS) and Cox proportional-hazards models were utilized to analyze the relationship between ACAG at CRRT initiation and ICU all-cause mortality of these patients. A total of 708 patients met the inclusion criteria in the study. The all-cause mortality of these patients during ICU hospitalization was 41.95%. Patients in the high ACAG group exhibited significantly higher ICU all-cause mortality rate than patients in the normal ACAG group (all *P* < 0.001). The Kaplan–Meier survival curves showed that the normal ACAG group had a higher ICU cumulative survival rate than the high ACAG group (log-rank test, *χ*_1_^2 ^= 13.620, *χ*_2_^2^ = 12.460, both *P* < 0.001). In the multivariate COX regression analyses, patients with higher ACAG (> 20 mmol/L) levels at the time of CRRT initiation in the MIMIC IV database and Huzhou Central Hospital were significantly correlated with ICU all-cause mortality after adjusting multiple potential confounding factors with hazard ratios of 2.852 (95% CI 1.718–4.734) and 2.637(95% CI 1.584–4.389), respectively. In critically AKI patients who undergo CRRT, higher ACAG (> 20 mmol/L) level at the initiation of CRRT was significantly correlated with ICU all-cause mortality. Therefore, clinicians should pay more attention to those patients with a higher ACAG value.

## Introduction

Acute kidney injury (AKI), characterized by a rapid weakening of kidney function, is a complex clinical disorder that carries a high societal, economic, and personal burden worldwide [1, 2]. Moreover, AKI is one of the most common reasons for an intensive care unit (ICU) admission and affects 33–66% of adult critically ill patients [3].

Generally, continuous renal replacement therapy (CRRT) is one of the most common interventions for AKI patients provided by intensivists in the ICU setting [4]. Even with medical advances, the mortality rate of AKI patients remains high, and there is currently no specific treatment that can reverse the progression of this disease [5]. Besides, critically ill patients with AKI who require CRRT have a high mortality rate [6]. Given the severity of AKI patients who need CRRT, it is necessary and important to have a way to predict the prognosis for these patients.

Acid–base disorders, especially for metabolic acidosis, are recognized as a frequent problem for critically ill patients and are associated with morbidity and mortality [7]. Generally, anion gap (AG), an indicator that reflects the unmeasured anion concentration, is applied to detect and evaluate the presence and severity of metabolic acidosis. However, because the albumin molecule carries a net negative charge, an increase or decrease in its concentration can affect the result of AG [8]. Hypoproteinemia, indeed, is very common in critically ill patients. Moreover, previous studies reported that hypoalbuminemia at the initiation of CRRT [9], as well as the dynamic changes of albumin during CRRT [10], are important risk factors for poor prognosis in AKI patients. Therefore, the formula of calculating the albumin corrected anion gap (ACAG) is described by Figge J et al. in 1998 to correct the AG for fluctuations in the albumin concentration [11, 12]. Prior studies have shown an association between elevated ACAG levels and poor outcomes of critically ill patients, such as sepsis [13] and cardiopulmonary arrest [14] etc.

To our knowledge, however, there have been no reports concerning the correlation between ACAG level and their prognosis in AKI patients who requires CRRT. We therefore initiated this study to determine whether there is an association between ACAG levels at the initiation of CRRT treatment and mortality among these patients.

## Materials and methods

### Source of data

The Medical Information Mart for Intensive Care-IV (MIMIC-IV, version 1.0) database is an openly accessible critical care database, and it provided the data with pre-existing institutional review board approval. This database contained data of 76,540 ICU admissions between 2008 and 2019 at the Beth Israel Deaconess Medical Center in Boston. One author was approved to exploit data from this database after completed the online course and examination (ZL, certification number: 36142713). Besides, Huzhou Central Hospital is a 1500-bed urban teaching hospital with an ICU census of around 900 annually, and the other part of our dataset were collected from the electronic hospitalization databases of this hospital. Our study was conducted according to the Declaration of Helsinki and approved by the Ethics Committee of Huzhou Central Hospital (Approval no: 202203021-01). Owing to the retrospective nature of this study, patient’s consent was not required. This study was a retrospective observational study, and it is reported based on the Strengthening the Reporting of Observational Studies in Epidemiology (STROBE) guidelines.

### Study population

Adult patients diagnosed with AKI and treated with CRRT in the ICU from the MIMIC-IV database (America, from 2008 to 2019) and Huzhou Central Hospital (China, from July 2019 to March 2022) were retrospectively enrolled. Patients were included if they fulfilled the Kidney Disease: Improving Global Outcomes (KDIGO) criteria for AKI in the first 7 day of their ICU admission. The AKI severity stage was also determined by the KDIGO guideline. If the patients had multiple ICU stay records, we only took the first ICU admissions. The exclusion criteria were the following: (1) age < 18 years, (2) end-stage renal disease, (3) discharged within 24 h after being admitted to ICU, (4) patients with missing data or inadequate information (such as AG, albumin, etc.).

### Study variables

Structured query language with PostgreSQL 10.13 was used for acquiring the data from the MIMIC-IV database. For each patient, we collected the demographics, clinical characteristics, comorbidities at the time of ICU admission, and laboratory values were collected at the time of CRRT initiation (detailed information is given in Table 1).

**Table 1:** Baseline characteristics of the study populations

Variables	MIMIC IV database (*n* = 452)				Huzhou central hospital (*n* = 256)			
	Survivors (*n* = 252)	Non-survivors (*n* = 200)	*t/z/χ*^*2*^	*P*	Survivors (*n* = 159)	Non-survivors (*n* = 97)	*t/z/χ*^*2*^	*P*
Age (years)	59.23 ± 15.15	60.27 ± 15.43	−0.722	0.471	66.19 ± 16.11	67.65 ± 15.89	−0.708	0.480
Female, *n *(%)	95 (37.70)	83 (41.50)	0.675	0.411	54(33.96)	31 (31.96)	1.721	0.423
SOFA score	12.64 ± 3.79	14.03 ± 4.08	−3.732	< 0.001	9.32 ± 3.86	11.82 ± 4.37	−4.785	< 0.001
Albumin (g/dl)	2.91 ± 0.76	2.78 ± 0.77	1.841	0.066	3.05 ± 0.57	2.89 ± 0.58	2.131	0.034
Anion gap (mmol/L)	20.94 ± 6.27	25.56 ± 8.16	−6.807	< 0.001	17.79 ± 6.82	20.87 ± 7.50	−3.373	0.001
ACAG (mmol/L)	24.66 ± 6.11	29.61 ± 8.29	−7.318	< 0.001	21.16 ± 6.56	24.64 ± 7.22	−3.954	< 0.001
WBC (×10^9^/L)	13.30(9.00, 20.10)	14.55 (9.55, 20.60)	−0.606	0.545	12.40 (7.70,17.80)	15.10 (8.00,23.00)	−1.486	0.137
Hemoglobin (g/L)	95.86 ± 17.83	92.97 ± 19.52	1.641	0.102	109.14 ± 28.35	99.00 ± 32.55	2.624	0.009
RDW (%)	16.92 ± 2.73	17.32 ± 3.37	−1.387	0.166	14.45 ± 2.09	15.07 ± 2.95	−1.955	0.052
Platelet (×10^9^/L)	121.50 (71.50, 197.00)	100.00 (62.00, 158.50)	3.082	0.002	121.00 (76.00, 189.00)	127.00 (58.00, 184.00)	0.164	0.870
Scr (umol/L)	327.08 (212.16, 433.16)	274.04 (203.32, 362.44)	3.329	0.001	315.90 (196.50, 460.60)	251.40 (187.50, 355.50)	2.254	0.024
BUN (umol/L)	18.87 (12.10, 29.01)	16.20 (9.97, 26.17)	2.147	0.032	20.43 (13.36, 30.92)	18.60 (11.89, 31.55)	0.718	0.473
Glucose (mmol/L)	7.50 (5.97, 9.72)	7.47 (5.83, 10.30)	0.102	0.919	7.85 (5.42,12.51)	9.56 (5.52, 13.84)	−0.996	0.319
Sodium (mmol/L)	136.62 ± 5.75	137.55 ± 6.96	−1.542	0.124	138.38 ± 9.08	143.38 ± 8.35	−4.408	< 0.001
Potassium (mmol/L)	4.54 ± 0.96	4.75 ± 1.01	−2.201	0.028	4.71 ± 1.06	4.51 ± 1.08	1.389	0.166
Chloride (mmol/L)	100.87 ± 6.76	100.09 ± 7.25	1.179	0.239	107.30 ± 10.07	109.44 ± 9.35	−1.691	0.092
Magnesium (mmol/L)	0.92 ± 0.19	0.95 ± 0.19	−1.581	0.115	0.82 (0.65, 1.01)	0.88 (0.73, 1.05)	−1.131	0.258
Total calcium (mmol/L)	2.06 ± 0.28	2.04 ± 0.31	0.554	0.580	1.97 ± 0.22	1.94 ± 0.20	1.143	0.254
Comorbidities								
* n*(%)	77 (30.56)	65 (32.50)	0.196	0.658	67 (42.14)	41 (42.27)	< 0.001	0.984
Hypertension	90 (35.71)	53 (26.50)	4.377	0.036	43 (27.04)	26 (26.80)	0.002	0.967
Diabetes	88 (34.92)	55 (27.50)	2.839	0.092	32 (20.13)	23 (23.71)	0.459	0.498
Atrial fibrillation	17 (6.75)	21 (10.50)	2.041	0.153	19 (11.95)	13 (13.40)	0.116	0.733
Cerebral infarction	15 (5.95)	27 (13.50)	7.536	0.006	9 (5.66)	24 (24.74)	19.536	< 0.001
Cardiac arrest	164 (65.08)	134 (67.00)	0.183	0.669	60 (37.74)	78 (80.41)	44.160	< 0.001
LOS ICU (days)	12.02 (6.22, 19.10)	5.56 (2.47, 12.18)	6.996	0.001	8.00 (6.00, 15.00)	5.00 (2.00, 15.00)	2.809	0.005

*SOFA* sequential organ failure assessment,* ACAG* albumin corrected anion gap,* WBC* white blood cell, RDW red cell distribution width, Scr serum creatinine; *BUN* Blood urea nitrogen, *ICU* intensive care unit, *LOS* *ICU* length of *ICU* stay

The normal reference value of AG in the MIMIC-IV database and the data from our hospital ranged from 8.00 to 20.00 mmol/l and 8.00 to 16.00 mmol/l, respectively. The ACAG was calculated with the following formula: AG (mmol/l) = (sodium + potassium)− (chloride + bicarbonate); ACAG = AG + {4.4-[albumin(g/dl)]}×2.5. The participants were divided into the survival group and the death group based on their survival during the ICU stay. Further, the study population was split into 2 groups based on their ACAG level using criteria from previous literature [15]: the normal ACAG group (ACAG 12–20 mmol/L) and high ACAG group (ACAG > 20 mmol/L). The primary endpoint was ICU all-cause mortality.

### Statistical analysis

Continuous data were expressed as mean ± standard deviation (SD) for normal distributions or median (interquartile range, IQR) for skewed distributions. Categorical data were described as numbers (%). The data were analyzed with one-sample *t* test, Wilcoxon rank-sum test or Chi-squared test as appropriate. Variables with a *P* value < 0.20 in the univariate analysis were included in the multivariate regression analysis. Kaplan–Meier curve was established to visualize differences in survival and was compared using the log-rank test. Restricted cubic spine (RCS) was utilized to calculate the correlation between ACAG and the risk of ICU all-cause mortality. Simultaneously, the association of ACAG with ICU all-cause mortality was determined using Cox proportional-hazards models and presented as hazard ratio (HR) with 95% confidence interval (CI). Stata 14.0 software and R language were used for all analyses. A two-sided *P* valueless than 0.05 was regarded as statistically significant.

## Results

### Study population

**Fig. 1:**
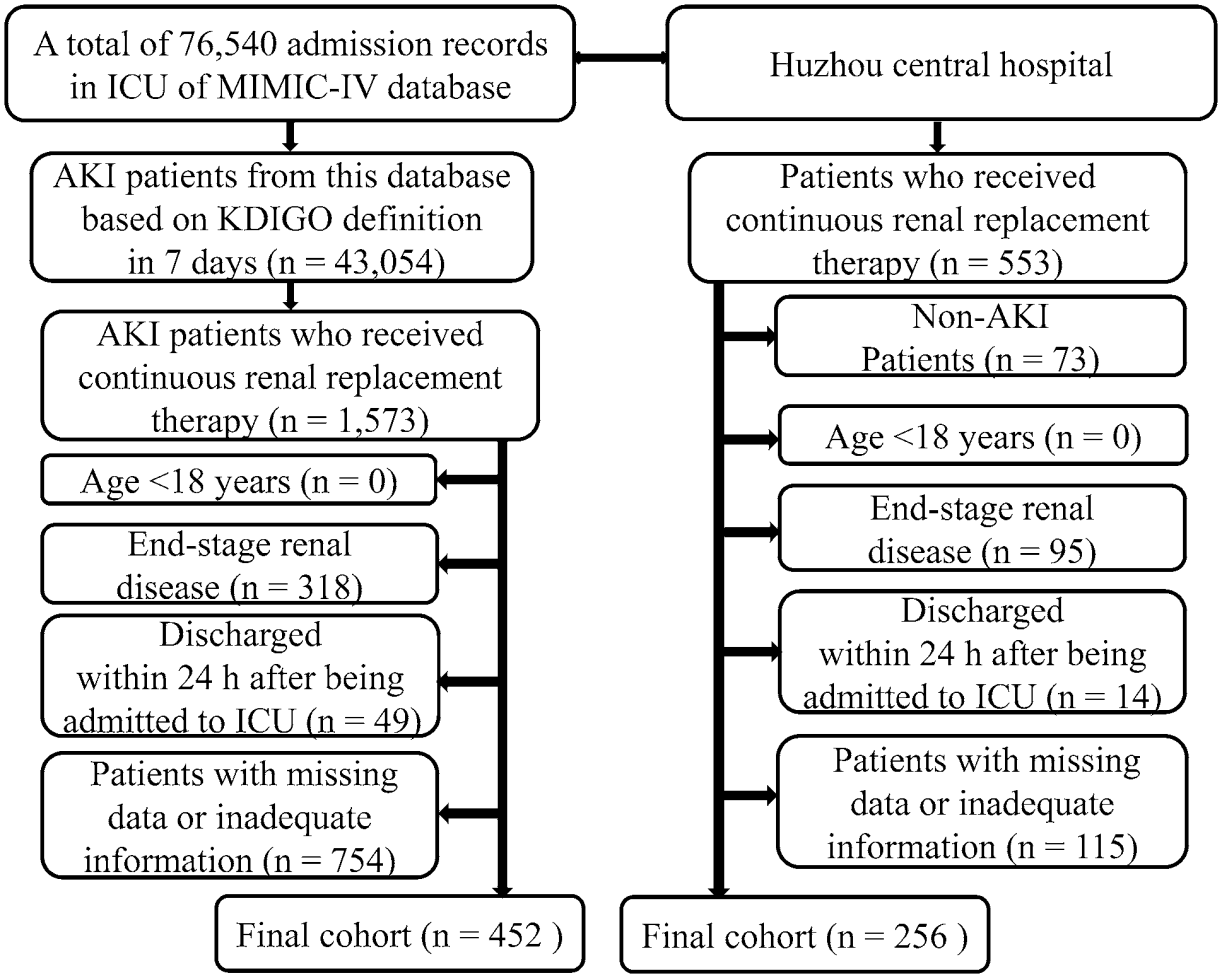
The flow chart of study subjects. *AKI* acute kidney injury, *ICU* intensive care unit, *KDIGO* kidney Disease improving global outcomes, *MIMIC* medical information mart for intensive care

The flowchart of included study population was displayed in Fig. 1. During the study period, a total of 452 and 256 eligible patients were ultimately enrolled from the MIMIC-IV database and Huzhou Central Hospital, respectively. The average age of patients from the MIMIC-IV database and our hospital were 59.69 ± 15.26 years and 66.74 ± 16.01 years. In both study populations, the SOFA score, anion gap, ACAG, and the incidence rate of cardiac arrest in the non-survivors group were significantly higher than those in the survivors group, while the serum creatinine level and length of ICU stay in the survivor group were higher than those in the non-survivors group (all *P* < 0.05). The baseline characteristics of the patients included are listed in Table 1.

### ACAG and all-cause mortality

As indicated in Table 2, the all-cause mortality rate during ICU hospitalization of the study population from the MIMIC IV database and our hospital is 44.25 and 37.89%, respectively. For the whole study population, the mortality rate of the high ACAG group (49.14%) is significantly higher than that of the normal ACAG group (21.31%, *χ*^2^ = 43.163, *P* < 0.001). Meanwhile, similar results are reported in both the subgroup of patients from the MIMIC IV database and our hospital (all *P* < 0.001).

**Table 2:** Comparison of ICU all-cause mortality between two groups

Group	MIMIC IV database (*n* = 452)				Huzhou Central Hospital(*n* = 256)				Overall (*n* = 708)			
	Survivors (*n* = 252)	Non-survivors (*n* = 200)	*χ*^*2*^	*P*	Survivors (*n* = 159)	Non-survivors (*n* = 97)	*χ*^*2*^	*P*	Survivors (*n* = 411)	Non-survivors (*n* = 297)	*χ*^*2*^	*P*
Normal ACAG	55 (76.39)	17 (23.61)			89 (80.18)	22 (19.82)			144 (78.69)	39 (21.31)		
High ACAG	197 (51.84)	183 (48.16)	14.785	< 0.001	70 (48.28)	75 (51.72)	27.193	< 0.001	267 (50.86)	258 (49.14)	43.163	< 0.001

ACAG albumin corrected anion gap, ICU intensive care unit

### Kaplan–Meier survival curve analysis

**Fig. 2:**
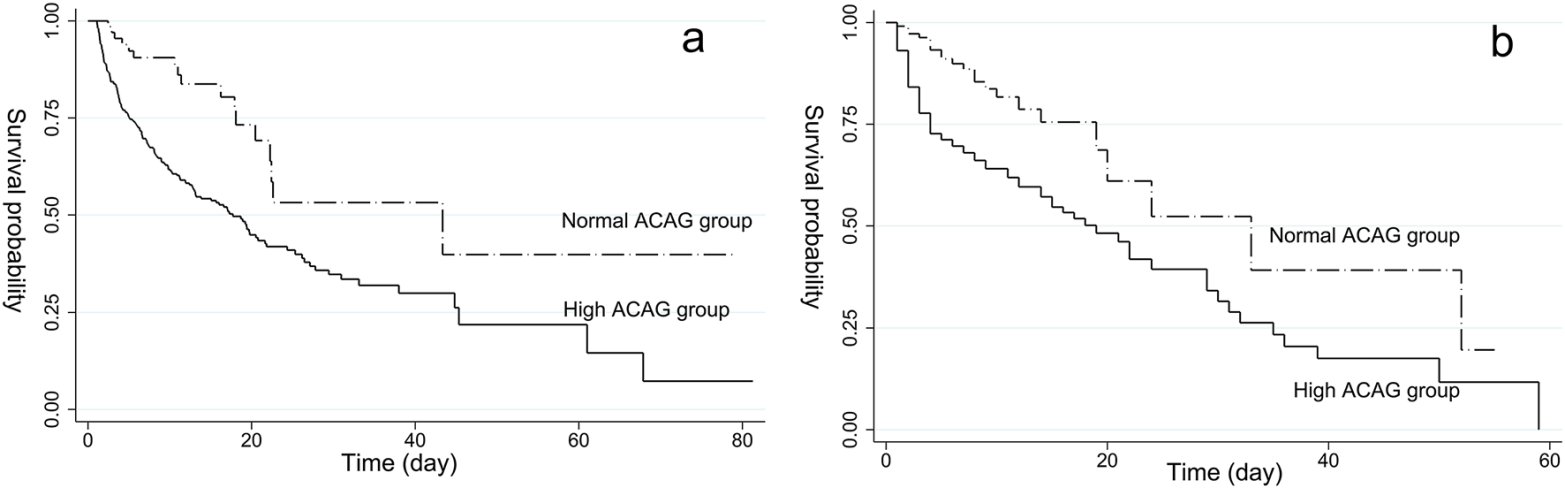
Kaplan–Meier curves of *ICU* all-cause mortality for the normal and high *ACAG* groups. **a** MIMIC IV database, **b** Huzhou Central Hospital. *ACAG* albumin corrected anion gap, *ICU* intensive care unit

In the Kaplan–Meier survival curves, as noted in Fig. 2, ICU cumulative survival rate is high for the normal ACAG group compared to the high ACAG group (log-rank test,* χ*_1_^2^ = 13.620, *χ*_2_^2^ = 12.460, both *P* < 0.001).

### Higher ACAG was significantly associated with ICU all-cause mortality

**Fig. 3:**
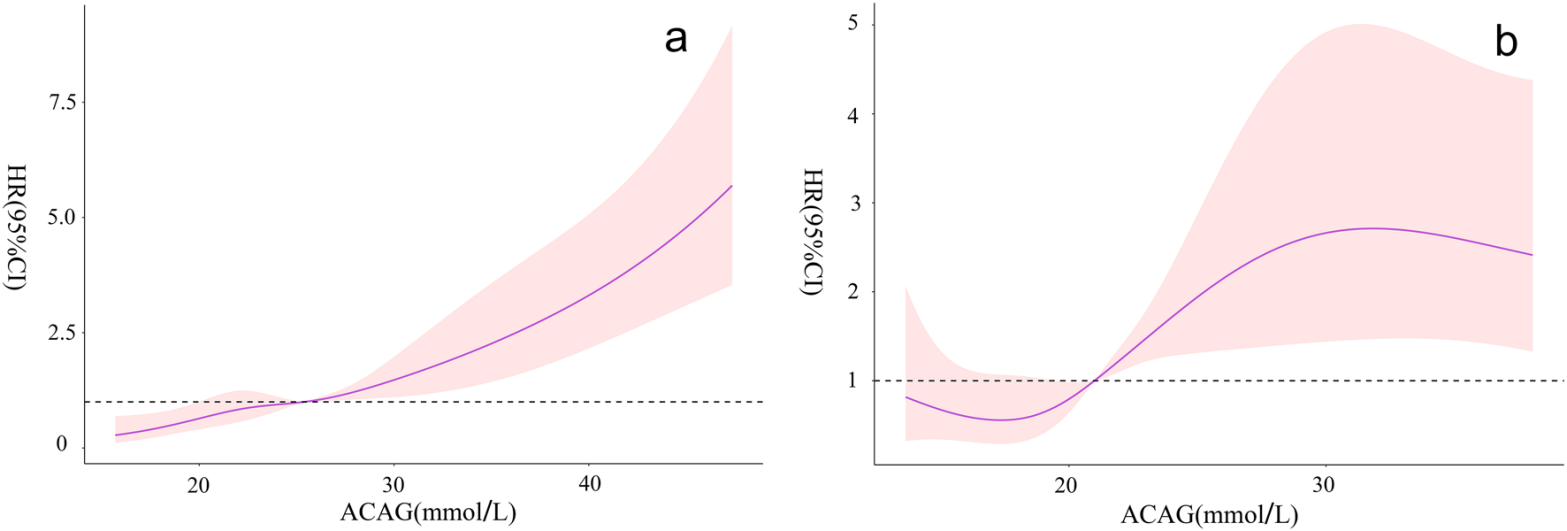
Association between *ACAG* at *CRRT* initiation and *ICU* all-cause mortality. **a** MIMIC IV database, **b** Huzhou Central Hospital *ACAG* albumin corrected anion gap, *CRRT* continuous renal replacement therapy, *ICU* intensive care unit

As noted in Fig. 3, a linear relationship is observed between ACAG at CRRT initiation and ICU all-cause mortality in the MIMIC IV database  = 1.380, *P* = 0.709), while the relationship in our hospital is nonlinear (*χ*^2^ = 9.640, *P* = 0.022).

For patients from the MIMIC IV database, the HR (95% CI) of ICU all-cause mortality for the high ACAG group compared to the normal ACAG group is 2.474 (1.504–4.069). The HR (95% CI) of ICU all-cause mortality for the high ACAG group compared to the normal ACAG group is 2.288 (1.416–3.696,) for patients from Huzhou Central Hospital. Based on the multivariate Cox proportional hazard analyses, an elevated ACAG at CRRT initiation (> 20 mmol/L) is an independent predictor for ICU all-cause mortality after adjusting for potential confounders (Table 3).

**Table 3:** Cox proportional hazard regression analysis for ICU all-cause mortality

Group	Non-adjusted model		Adjusted model	
	HR (95% CI)	*p* value	HR (95% CI)	*p* value
MIMIC IV database				
Normal ACAG	1		1	
High ACAG	2.474 (1.504–4.069)	< 0.001	2.852 (1.718–4.734)	< 0.001
Huzhou central hospital				
Normal ACAG	1		1	
High ACAG	2.288 (1.416–3.696)	0.001	2.637 (1.584–4.389)	< 0.001

The adjusted model of MIMIC IV database adjusted for SOFA score, hemoglobin, red cell distribution width, platelet, serum creatinine, blood urea nitrogen, magnesium, diabetes, atrial fibrillation, cerebral infarction and cardiac arrest

The adjusted model of Huzhou Central Hospital adjusted for SOFA score, white blood cell, hemoglobin, red cell distribution width, serum creatinine, acute respiratory failure and cardiac arrest

*ACAG* albumin corrected anion gap, *ICU* intensive care unit

## Discussion

The current study found that patients in the high ACAG group exhibited a higher ICU all-cause mortality rate than the normal ACAG group. Simultaneously, the Kaplan–Meier survival curves showed that ICU cumulative survival rate is high for the normal ACAG group compared to the high ACAG group. The results of the RCS were different, which might be a result of the differences in race of patients, sample sizes, and normal reference range of AG, etc. Furthermore, compared with participants with normal ACAG levels, those with high ACAG levels (> 20 mmol/L) in the MIMIC-IV database and in our hospital denoted a 1.85-fold and 1.64-fold increased risk of ICU all-cause mortality, respectively. Hence, clinicians ought to pay more attention to those patients with ACAG more than 20 mmol/L because of the poor prognosis associated with high ACAG levels.

It is universally acknowledged that acid–base homeostasis is the basis of life, and several organ systems including brain, lungs, kidney, and liver are involved in the regulation of acid–base balance [16, 17]. Indeed, the kidneys play a central role in maintaining electrolyte homeostasis and acid–base balance [18]. AKI is a common and severe complication in patients hospitalized in the ICU [19], characterized by an increase in SCr levels and impairment of kidney functions such as fluid, electrolyte, and acid–base balance [20]. Moreover, metabolic acidosis is one of the most common complications in AKI patients [21]. Accumulating evidence suggested that metabolic acidosis is associated with an increased risk of chronic kidney disease progression [22, 23], as acidosis can reduce renal blood flow and increase the release of inflammatory mediator [24]. However, studies on the relationship between metabolic acidosis and the progression of kidney dysfunction during AKI are scarce. One experimental study [25] demonstrated that metabolic acidosis exacerbates renal injury through the high expression of NF-κB in an ischemia/reperfusion-induced AKI model. Besides, previously published articles indicated that the indicators of metabolic acid–base status, such as AG, base excess, and lactate, etc., can serve as potential prognostic predictors for AKI patients.

Metabolic acidosis is one of the most common complications of patients hospitalized in ICU, and high AG metabolic acidosis is one subcategory of metabolic acidosis [26, 27]. In a prospective study [28], plasma AG was measured in 500 critically ill patients, and the authors concluded that an elevated AG level on ICU admission was associated with higher mortality and longer length of stay in the ICU. In addition, several other studies have also explored the relationships between metabolic acidosis and clinical outcomes of AKI patients. Cheng Y et al. [29] found a U-shaped association between base excess value measured at the ICU admission and 30 day all-cause mortality in critically AKI patients, and both lower (≤ − 3 mEq/L)and higher (≥ 9 mEq/L) base excess would increase the risk of 30 day all-cause mortality. One latest study by Uusalo and colleagues [30] acknowledged that for perioperative AKI patients requiring CRRT, blood lactate at ICU admission and CRRT initiation were both independently associated with mortality.

ACAG, which is combined serum albumin and AG, can be used to distinguish acidosis caused by acid load or base deficit, which correspond to two pathological states of human body-hypoalbuminemia and metabolic acidosis [31]. Also, as mentioned previously, ACAG value is a more accurate predictor for metabolic acidosis for critically ill patient because of their hypoalbuminemia. Recently, one study has reported that low serum albumin before the initiation of CRRT is an independent predictor of mortality of AKI patients who underwent CRRT [9]. It’s worth noting that the incidence of hypoalbuminemia (albumin < 3.5 g/dL) is as high as 80.23% (568/708) in the overall patient population in our study. Cheng and colleagues observed a nonlinear association between AG measured at ICU admission and 30 day all-cause mortality, and high-AG levels (≥ 14 mmol/L) at the time of ICU admission are independently associated with 30, 90 and 365 day all-cause mortality in patients with AKI [32]. However, studies regarding the relationship between ACAG and mortality in patients undergoing CRRT for AKI have not been reported before. Consequently, we carried out this study and found that ACAG at the initiation of CRRT treatment is an independent risk factor that can predict the ICU all-cause mortality of these patients.

The current study has several strengths. First, this study is the first to explore the relationship between ACAG and mortality in patients undergoing CRRT for AKI. Second, the data in this study are collected from two distinct sources: one subtset of data is from a well-established registry of MIMIC-IV database with high data quality from America, and the other subset comes from a Chinese teaching hospital. Data from both subsets generate positive results that are consistent with each other based our analysis. In conclusion, our findings demonstrate elevated ACAG at CRRT initiation is associated with mortality of AKI patients treated with CRRT.

However, there were several limitations of our study. First, this study is a retrospective study, therefore, inherent biases cannot be ignored because of the retrospective nature. Second, due to the absence of data regarding out-of-hospital mortality in the MIMIC-IV (v1.0) database and Huzhou Central Hospital, we are unable to evaluate the relationship between ACAG and prognosis after discharge in AKI patients undergoing CRRT. Third, the current study explored the relationship between the ACAG and mortality based on a single ACAG measurement. Consequently, further study is warranted to investigate the relationship between the dynamic changes of ACAG and mortality of these patients.

In conclusion, the current evidence suggested that a higher ACAG level (> 20 mmol/L) at the initiation of CRRT treatment is associated with ICU all-cause mortality in critically AKI patients who undergo CRRT, and ACAG can serve as an early indicator of adverse outcomes for these patients. Nonetheless, additional studies, especially a rigorously designed prospective study, are required to evaluate and verify this finding.

## Data Availability

Not applicable.

## Abbreviations

ACAG: Albumin corrected anion gap
AG: Anion gap
RCS: Restricted cubic spine
AKI: Acute kidney injury
CRRT: Continuous renal replacement therapy
CI: Confidence interval
HR: Hazard ratio
ICU: Intensive care unit
IQR: Interquartile range
KDIGO: Kidney Disease Improving Global Outcomes
MIMIC: Medical Information Mart for Intensive Care
Scr: Serum creatinine
SD: Standard deviation
